# Insulin DEgludec/LIraglutide versus multiple daily insulin injections in the transition from hospital to outpatient management assessed by continuous glucose monitoring: the DELI transition trial

**DOI:** 10.1007/s00125-025-06446-y

**Published:** 2025-08-04

**Authors:** Ana M. Gómez-Medina, Diana C. Henao-Carillo, Lina P. Villamil-Castañeda, Yaline Gómez-Quesada, Oscar M. Muñoz-Velandia, Carlos A. Yepes, Salma N. Chaim, Carlos E. Pertuz-Noriega, Pablo Aschner

**Affiliations:** 1 Endocrinology Unit, Pontificia Universidad Javeriana, Bogotá, Colombia; 2 Endocrinology Unit, Hospital Universitario San Ignacio, Bogotá, Colombia; 3 Department of Internal Medicine, Pontificia Universidad Javeriana, Bogotá, Colombia; 4 Department of Internal Medicine, Hospital Universitario San Ignacio, Bogotá, Colombia; 5 School of Medicine, Pontificia Universidad Javeriana, Bogotá, Colombia; 6 School of Medicine, Hospital Universitario San Ignacio, Bogotá, Colombia

**Keywords:** Colombia, Hospital to home transition, IDegLira, Type 2 diabetes mellitus

## Abstract

**Aims/objective:**

The aim of the study was to assess the safety profile (defined as the percentage of patients with at least one hypoglycaemic event [more than 15 min with glucose levels <3.0 mmol/l as documented by continuous glucose monitoring] in the first 4 weeks of follow-up) for insulin degludec/liraglutide (IDegLira) compared with multiple daily insulin injections (MDI) during the transition from hospital to an outpatient setting.

**Methods:**

The study was an open-label, randomised, controlled clinical trial comparing IDegLira to MDI after hospital discharge in patients with type 2 diabetes. The study evaluated the percentage of patients with at least one hypoglycaemic event, the hypoglycaemia event density, the time in range (TIR 3.8–10 mmol/l), the time below range (TBR <3.0 or <3.8 mmol/l), and other glycaemic management metrics measured by continuous glucose monitoring.

**Results:**

Sixty-four patients were included in the analysis (32 in each group). They had a baseline HbA_1c_ of 103 ± 11.6 mmol/mol (11.6 ± 1.7%) and age of 58 ± 12.4 years (means ± SD). The proportion of patients with at least one hypoglycaemic event (plasma glucose <3.0 mmol/l) was lower in the IDegLira group than in the MDI group (6.2% vs 31.3%; *p*<0.010), as was the hypoglycaemia event density (incidence rate ratio 15.2; 95% CI 6.2, 48.2; *p*<0.001), TBR <3.8 mmol/l (0.9% vs 2.9%; *p*=0.019) and TBR <3.0 mmol/l (0.6% vs 1.3%, *p*=0.008). The TIR 3.8–10 mmol/l was higher in the IDegLira group (80.6% vs 69.7%; *p*=0.008). The findings were consistent regardless of baseline HbA_1c_.

**Conclusions/interpretation:**

IDegLira proved to be safer and more effective than MDI for individuals with type 2 diabetes who had suboptimal glycaemic control, aiding in their transition from hospital to outpatient care.

**Trial registration:**

Clinicaltrials.gov NCT05767255

**Funding:**

This research was funded by a grant from the Asociación Colombiana de Endocrinología, Diabetes y Metabolismo (ACE).

**Graphical Abstract:**

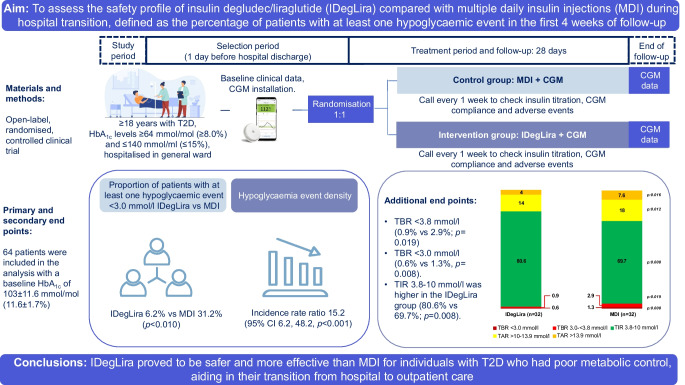

**Supplementary Information:**

The online version contains peer-reviewed but unedited supplementary material available at 10.1007/s00125-025-06446-y.


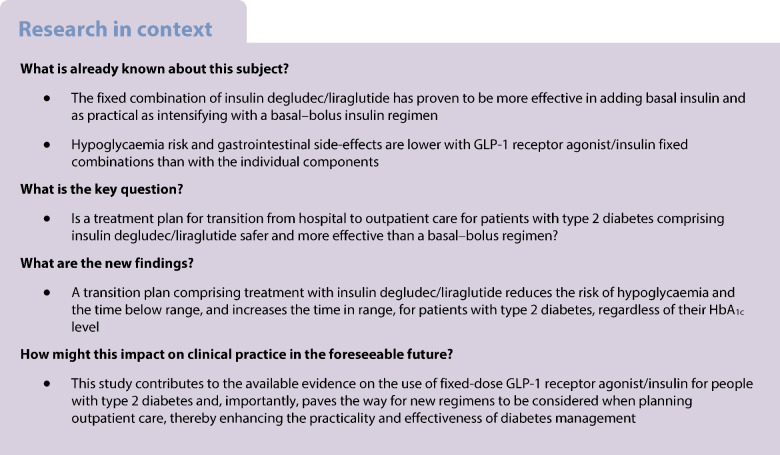


## Introduction

Individuals with type 2 diabetes experience hospitalisation rates that are two to six times higher than patients without diabetes, with readmission rates that are almost double. During hospitalisation, insulin requirements may fluctuate significantly, often resulting in a discharge insulin regimen that may be overly aggressive, thus increasing the risk of readmission and severe hypoglycaemia within the first 30 days after discharge [1, 2]. There is also an increased risk of weight gain in patients who are discharged from hospital on multiple daily insulin injections (MDI). These factors, combined with the burden of MDI, can hinder treatment adherence [3]. Additionally, up to 30% of type 2 diabetes patients who continued MDI after hospitalisation reported at least one hypoglycaemic event <3.8 mmol/l within 1 week after discharge [2]. As such, it is essential to adjust the basal insulin dose at discharge, and carefully evaluate the need for prandial insulin on a case-by-case basis.

The combination of insulin degludec and liraglutide (IDegLira; Novo Nordisk) has proven helpful in intensifying the treatment of individuals with type 2 diabetes. It enables better glycaemic control than use of each component individually, with a lower risk of hypoglycaemia and fewer gastrointestinal side-effects [4]. A recent controlled clinical trial demonstrated similar efficacy in reducing HbA_1c_ compared with an MDI regimen in patients with HbA_1c_ ≥86 mmol/ml (≥10.0%) [3]. This therapy simplifies management, reducing hypoglycaemic events by more than 50% in outpatient settings [5]. However, to our knowledge, no clinical studies have yet evaluated its use during the transition from hospital to outpatient care.

This randomised, open-label clinical trial is designed to evaluate whether using a fixed combination treatment (IDegLira) is safer than an MDI regimen for adults with type 2 diabetes after transition from hospital to outpatient care. The primary safety measure is the percentage of patients who experience at least one hypoglycaemic event detected by continuous glucose monitoring (CGM) in the first 28 days after discharge.

## Methods

This is an open-label randomised clinical trial. Inclusion criteria were patients aged over 18 years with a diagnosis of type 2 diabetes, with HbA_1c_ levels ≥64 mmol/mol (≥8.0%) and ≤140 mmol/ml (≤15%), who were hospitalised in a general ward, and who required MDI during hospitalisation according to hospital guidelines and were eligible for MDI therapy at the time of transition from hospital to outpatient care.

All patients included in this study were Hispanic. The patients were treated at the Hospital Universitario San Ignacio (Bogotá, Colombia), between November 2022 and June 2024. Patients with an eGFR <15 ml/min per 1.73 m^2^ determined using the CKD-EPI equation [6], a history of acute or chronic pancreatitis in the last 3 months, hypersensitivity to any components of the IDegLira combination, pregnant or breastfeeding women, individuals receiving treatment for obesity with glucagon-like peptide-1 (GLP-1) receptor agonists, previous MDI therapy with a total daily dose greater than 70 units/day or less than 10 units/day, or steroid-associated hyperglycaemia were excluded. Consent was obtained from every patient, and this study was approved by the institutional ethics committee (Hospital Universitario San Ignacio, FM-CIE-0738-22). The study was registered at ClinicalTrials.gov (NCT05767255) under the name ‘Risk of Hypoglycemia in the Transition From Inpatient to Outpatient Setting. Comparative Study of Basal-bolus Insulin Versus Basal Insulin Plus GLP-1 Analogue’ (also referred to as the DEgludec/LIraglutide transition trial [DELI transition trial]), and is reported according to the Consolidated Standards of Reporting Trials (CONSORT) updated guidelines for reporting parallel-group randomised trials.

Once a patient was selected to participate, the researcher contacted a central facility where the patient was randomly allocated to one of two treatment groups (allocation by central office). Randomisation was performed by an independent staff member who was blinded to patient characteristics, using a simple randomisation numerical sequence generated by a random number generator. The researcher could not change the assigned treatment. The intervention group was treated with IDegLira, starting at 10 IU if no insulin was used prior to hospitalisation or at 16 IU if insulin was previously used at any dose, starting on day 1 after hospital discharge. In the control group, the total daily dose required at the end of hospitalisation was reduced by 20% to calculate the insulin dose to be used for outpatient treatment. In the case of persistent hyperglycaemia symptoms (>13.3 mmol/l) for more than 3–5 days or symptoms of catabolism (polydipsia, polyuria or polyphagia) without resolution in outpatient follow-up, the protocol suggested that the patient should transition to an MDI regimen. Initiation of other glucose-lowering medications after discharge was delayed until patients had completed the 4-week follow-up period. Participants were not blinded to group assignment given the nature of the interventions (one vs multiple doses daily).

In both groups, follow-up was performed via CGM using a FreeStyle Libre device (Abbott) installed at patient discharge. During the initial evaluation at the time of discharge, all patients received a 60 min individual session with a diabetes educator. The session covered the technique of insulin delivery and titration, the detection and management of hypoglycaemia, and the proper use of the CGM. Patients were subsequently contacted weekly via telephone to gather information about adverse effects, insulin titration and hypoglycaemia events. During follow-up, patients were instructed to titrate the fixed dose of IDegLira in the intervention group, and basal and preprandial insulin in the control group, according to the DUAL VII study protocol (see electronic supplementary material [ESM] Tables 1–3). In week 4, an in-person review was held to download CGM data, and measurements of blood pressure and anthropometric variables were performed for comparison of body weight and BMI by treatment group.

The primary outcome was the percentage of patients who experienced at least one hypoglycaemic episode (more than 15 min with glucose levels 3.0 mmol/l as documented by CGM) within the first 4 weeks after hospital discharge. Secondary outcomes included CGM metrics such as time in range (TIR, 3.8–10 mmol/l [70–180 mg/dl]), CV, time above range (TAR, >10 mmol/l [>180 mg/dl] or >13.9 mmol/l [>250 mg/dl]) and the glucose management indicator (GMI). Severe hypoglycaemic episodes were defined according to clinical practice guidelines [7]. Additionally, composite outcomes were evaluated, such as the percentage of patients with a TIR >70% without episodes of hypoglycaemia below 3.8 mmol/l or 3.0 mmol/l), and mean glucose <8.5 mmol/l without hypoglycaemia episodes <3.0 mmol/l [8].

Based on data from the DUAL VII study, a sample of 64 patients (32 per group) was estimated as necessary to detect a 61% reduction in the percentage of patients with at least one episode of hypoglycaemia, as observed in the IDegLira group (20%) compared with the MDI group (53%), with an alpha error of 0.05 and a power of 80%.

For statistical analysis, continuous variables with a normal distribution were compared using Student’s unpaired *t* test, while non-normally distributed variables were compared using the Mann–Whitney *U* test. The Shapiro–Wilk test was used to assess the assumption of normality. Categorical variables were compared using a χ^2^ test. The analyses followed an intention-to-treat approach. Hypoglycaemia incidence was assessed by calculating the relative risk of experiencing at least one hypoglycaemic episode. The incidence density was evaluated by comparing the number of hypoglycaemic episodes per patient-day in each group, with the results presented as an incidence rate ratio. No adjustment was made for multiple comparisons. A subgroup analysis examined glycaemic management outcomes based on baseline HbA_1c_ levels and prior therapy type before hospitalisation. Statistical analysis was performed using Stata version 16 (StataCorp).

## Results

**Fig. 1:**
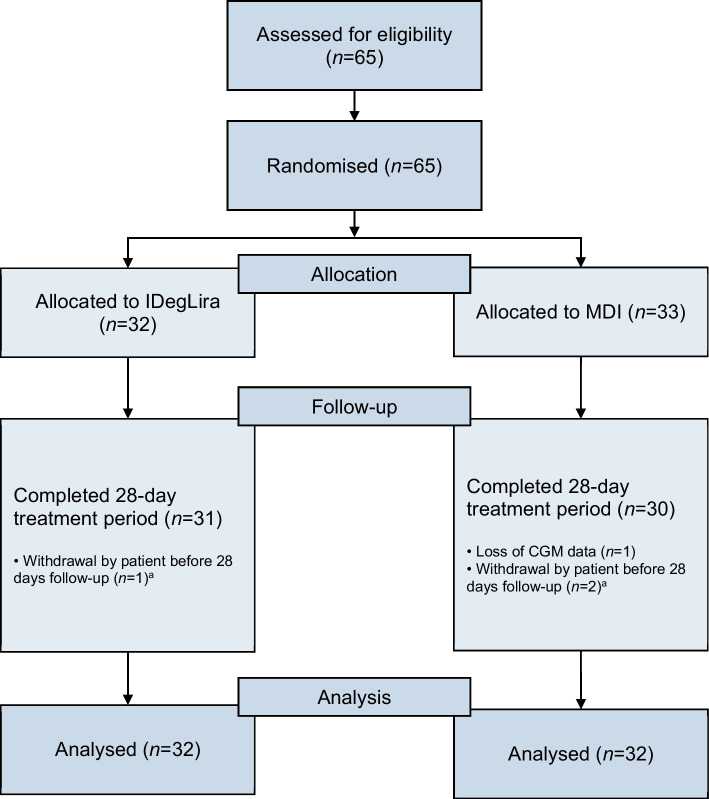
Flow chart for participant inclusion. ^a^For the patients who were lost to follow-up at 28 days, the information available from the first 14 days was taken into account

A total of 65 patients were randomised for inclusion in the study, with 32 assigned to the IDegLira group and 33 to the MDI group; one patient was excluded from the final analysis due to missing CGM data. The participants had a mean age of 58 ± 12.4 years. All of them were Hispanics. The primary reason for hospitalisation was infection (35.7%), followed by suboptimal diabetes control (35%) and cardiovascular disease (7.1%). The mean baseline HbA_1c_ was 103 ± 11.6 mmol/mol (11.6 ± 1.7%), with no significant difference between groups. However, a higher proportion of patients in the MDI group had a history of coronary heart disease compared with the IDegLira group (12.5% vs 0%; *p*=0.039). Other characteristics were similar across groups (Table 1). The flow chart for participant inclusion is shown in Fig. 1.

**Table 1:** Comparison of patient characteristics for the two treatment groups

Variable	IDegLira (*N*=32)	MDI (*N*=32)	*p* value
Age, years	57 ± 12.4	59 ± 10.5	0.759
Sex (male)	17 (53.1)	14 (43.8)	0.453
BMI (kg/m^2^)			
<25	7 (21.9)	12 (37.5)	0.337
25–30	17 (53.1)	12 (37.5)	
>30	8 (25.0)	8 (25.0)	
Duration of diabetes, years	5.0 (0–10.5)	7.1 (0–12.5)	0.503
Previous treatment^a^			
Basal insulin	6 (18.8)	6 (18.8)	0.714
Basal–bolus	3 (9.4)	5 (15.6)	
Metformin	8 (25.0)	7 (21.9)	
DPP4i	0 (0)	1 (3.1)	
Sulfonylurea	1 (3.1)	0 (0)	
SGLT2i	1 (3.1)	0 (0)	
None	14 (43.8)	12 (37.5)	
Initial HbA_1c_, mmol/mol	103 ± 11.6	103 ± 11.6	0.961
Initial HbA_1c_, %	11.6 ± 1.7	11.6 ± 1.7	0.961
Creatinine, μmol/l	71.6 (59.2–84)	72.5 (57.5–79.6)	0.812
eGFR, ml/min per 1.73 m^2^			
<60	3 (9.4)	3 (9.4)	1.000
60–90	7 (21.9)	7 (21.9)	
>90	22 (68.8)	22 (68.8)	
Diabetes-related complications			
Neuropathy	0 (0)	0 (0)	
Retinopathy	3 (9.4)	3 (9.4)	1.000
Chronic kidney disease	5 (15.6)	6 (18.8)	0.740
Heart failure	1 (3.1)	4 (12.5)	0.162
Coronary heart disease	0 (0)	4 (12.5)	0.039
Stroke	1 (3.1)	0 (0)	0.313
Peripheral artery disease	1 (3.1)	2 (6.3)	0.554
Hypertension	16 (50.0)	18 (56.3)	0.616
Insulin requirement during hospitalisation (units/day)			
Basal insulin	20.9 ± 7.0	20.9 ± 6.6	1.000
Bolus insulin	6.4 ± 2.1	6.4 ± 2.0	1.000
TDD	27.1 ± 7.8	27.3 ± 7.9	0.919
Insulin required at end of follow-up (units/day)			
Basal insulin	21.8 ± 5.9	26.7 ± 7.6	0.005
Bolus insulin	NA	27.1 ± 8.2	NA
TDD	21.8 ± 5.9	53.8 ± 15.1	<0.001

Values are means ± SD or median (IQR) for continuous variables, and *n* (%) for categorical variables

^a^Patients may have been taking one or more medications to treat diabetes prior to hospitalisation

DPP4i, inhibitors of dipeptidyl peptidase 4; SGLT2i, sodium–glucose cotransporter-2; TDD: total daily dose; NA, not applicable

The IDegLira group had a lower percentage of patients with at least one hypoglycaemic episode <3.0 mmol/l detected by CGM (6.2% vs 31.3% for the MDI group; *p*=0.010). Similarly, the incidence density of hypoglycaemic events was 15 times higher in the MDI-treated group (0.086 vs 0.005 events/patient-day; incidence rate ratio 15.2; 95% CI 6.2, 48.2, *p*<0.001).

**Fig. 2:**
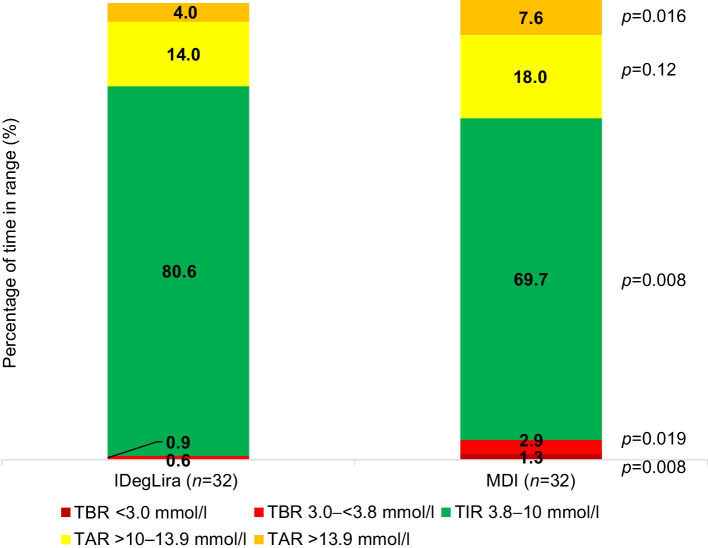
Glucose metrics obtained through CGM

The percentage time below range (TBR) for glucose levels <3.8 mmol/l (0.9% vs 2.9%; *p*=0.019) and <3.0 mmol/l (0.6% vs 1.3%, *p*=0.008) was significantly lower in the IDegLira group compared with the MDI group. The TIR 3.8–10 mmol/l was higher in the IDegLira group compared with the MDI group (80.6% vs 69.7%; *p*=0.008). The metabolic control metrics recorded during follow-up with CGM are shown in Fig. 2.

In the MDI group, the CV and the proportion of patients with a CV >36% (34.4% vs 9.4%; *p*=0.016) were higher than in the IDegLira group. The mean interstitial glucose was similar in the IDegLira group (8.4 ± 1.7 mmol/l) and the MDI group (8.3 ± 1.8 mmol/l), as was the mean GMI (51 ± 2.4 and 52 ± 2.4 mmol/mol, respectively).

The proportions of patients achieving a TIR 3.8–10 mmol/l >70% and TBR <3.0 mmol/l <1% were higher in the IDegLira group (Table 2), as was the proportion of patients with mean glucose <8.5 mmol/l without hypoglycaemia episodes <3.0 mmol/l (71.9% vs 43.8%; *p*=0.023). Additionally, more patients achieved over 70% TIR without level 1 (<3.8 mmol/l) and 2 (<3.0 mmol/l) hypoglycaemia episodes (71.9% vs 43.8%; *p*=0.023 and 78.1% vs 43.8%; *p*=0.005). During follow-up, patients in the IDegLira group lost weight compared to those treated with MDI (who gained weight) (−2.1 vs +1.0 kg; *p*<0.001).

**Table 2:** Comparison of metrics from FreeStyle Libre CGM by treatment group

	IDegLira (*N*=32)	MDI (*N*=32)	*p* value
Proportion of patients achieving treatment goals			
TIR 3.8–10 mmol/l >70%	25 (78.1)	17 (53.1)	0.035
TBR <3.8 mmol/l <4%	30 (93.8)	23 (71.9)	0.020
TBR <3.0 mmol/l <1%	30 (93.8)	22 (68.8)	0.010
TAR >10 mmol/l <25%	26 (81.3)	22 (68.8)	0.024
TAR >13.9 mmol/l <4%	26 (81.3)	18 (56.3)	0.031
Mean glucose <8.5 mmol/l and TBR 3.0 mmol/l <1%	23 (71.9)	14 (43.8)	0.023
TIR 3.8–10 mmol/l >70% and TBR <3.8 mmol/l <4%	23 (71.9)	14 (43.8)	0.023
TIR 3.8–10 mmol/l >70% and TBR <3.0 mmol/l <1%	25 (78.1)	14 (43.8)	0.005
CV %	28 (22.3–33.1)	34 (30–38.6)	<0.001
CV >36%	3 (9.4)	11 (34.4)	0.016
GMI, mmol/mol	49 (43–53)	52 (45–58)	0.161
GMI, %	6.6 (6.1–7)	6.9 (6.3–7.5)	
Glucose, mmol/l	7.7 (6.6–8.8)	8.3 (6.9–9.7)	0.151
Sensor time use, %	85.8 (81.5–94.5)	83.2 (79–92.5)	0.536
Body weight change, kg	−2.1 ± 3.5	1.0 ± 3.4	<0.001

Values are means ± SD or median (IQR) for continuous variables, and *n* (%) for categorical variables. Results are reported according to the CGM consensus [8]

Subgroup analysis based on previous therapy showed a more significant proportion of patients achieving TIR >70% when previously managed with oral therapies only (88.9% vs 33.3%; *p*=0.016) (Table 3). Additionally, more of the patients previously managed with insulin reached the goal of TBR <3.8 mmol/l or <3.0 mmol/l when subsequently receiving IDegLira management (100% vs 63.6%; *p*=0.043). Fifty-six patients (87.5%) included in the study had an HbA_1c_ >86 mmol/mol (>10%) at randomisation, of whom 78.6% reached the TIR target with IDegLira, compared with only 46.4% of the MDI group (*p*=0.013).

**Table 3:** Comparison of glycaemic outcomes based on prior treatment and HbA_1c_

Proportion of patients achieving treatment goals	IDegLira (*N*=32)	MDI (*N*=32)	*p* value
Prior treatment			
TIR 3.8–10 mmol/l >70%			
Oral=18	8 (88.9)	3 (33.3)	0.016
Insulin=18	5 (55.0)	6 (54.5)	0.964
None=26	12 (85.7)	8 (66.7)	0.250
TBR 3.8–10 mmol/l <4%			
Oral=18	8 (88.9)	8 (88.9)	1.000
Insulin=18	9 (100)	7 (63.6)	0.043
None=26	13 (92.9)	11 (91.7)	0.910
TBR <3.0 mmol/l <1%			
Oral=18	9 (100)	8 (88.9)	0.303
Insulin=18	9 (100)	7 (63.6)	0.043
None=26	14 (100)	11 (91.7)	0.271
Baseline HbA_1c_			
TIR 3.8–10 mmol/l >70%			
<86 mmol/mol (<10%)	4 (100)	4 (100)	
>86 mmol/mol (>10%)	22 (78.6)	13 (46.4)	0.013
TBR 3.8–10 mmol/l <4%			
<86 mmol/mol (<10%)	4 (100)	4 (100)	
>86 mmol/mol (>10%)	26 (92.9)	22 (78.6)	0.127
TBR <3.0 mmol/l <1%			
<86 mmol/mol (<10%)	4 (100)	3 (75.0)	0.285
>86 mmol/mol (>10%)	28 (100)	23 (82.1)	0.019

Values correspond to *n* (%). The percentages were calculated with respect to the number of patients in the category shown in each row, presented differently for each treatment

None of the patients treated with IDegLira had to be switched to MDI. The proportion of patients experiencing no side-effects was similar for both groups (IDegLira 87.6% vs MDI 96.9%; *p*=0.165). However, there were 26 events of severe hypoglycaemia during follow-up in the MDI group (Table 4). A higher proportion of patients experienced gastrointestinal adverse events (6.3% vs 3.1%; *p*=0.555) and symptoms of asthenia and adynamia (3.1% vs 0%; *p*=0.315). One patient in the IDegLira group experienced an acute kidney injury. This was subsequently resolved.

**Table 4:** Comparison of adverse events between groups at the end of the follow-up

Adverse event	IDegLira (*N*=32)	MDI (*N*=32)	*p* value
Number of events of severe hypoglycaemia^a^	0	26	
Number of patients with ≥1 event of severe hypoglycaemia	0 (0)	10 (31.3)	<0.001
Number of patients with gastrointestinal events^b^	2 (6.3)	1 (3.1)	0.555
Number of patients with asthenia and adynamia	1 (3.1)	0 (0)	0.315
Acute renal injury event^c^	1 (3.1)	0 (0)	0.315

Data are *n* or *n* (%)

^a^Severe hypoglycaemia comprised altered mental and/or physical status requiring assistance for treatment of the hypoglycaemia, irrespective of glucose level

^b^Gastrointestinal events were mainly nausea and vomiting

^c^This was a KDIGO 1 event according to the Kidney Disease: Improving Global Outcomes classification

## Discussion

This randomised clinical trial demonstrates that, for patients who are transitioning from inpatient to outpatient care, IDegLira treatment leads to a lower incidence of hypoglycaemic events and reduces time spent in hypoglycaemia <3.8 mmol/l or <3.0 mmol/l. This results in improved metabolic control as measured by the percentage of TIR achieved. Additionally, we observed a lower proportion of patients experiencing hypoglycaemia and a decrease in hypoglycaemic event frequency, as measured by the incidence density ratio.

To our knowledge, this study is the first to evaluate the safety and efficacy of CGM during the transition from inpatient to outpatient care in patients with baseline HbA_1c_ >86 mmol/mol (>10%). It shows that IDegLira is a simpler and safer strategy compared with MDI to achieve optimal glycaemic control, making it easier for the primary care team to continue with this therapy. In addition, there is strong evidence for the long-term efficacy of IDegLira in the outpatient setting [4, 9]. For example, the recent DUAL HIGH study demonstrated comparable efficacy in reducing HbA_1c_ with IDegLira compared with MDI therapy, with a significant reduction in hypoglycaemic events and less weight gain among patients with type 2 diabetes and HbA_1c_ ranging from ≥75 to 140 mmol/mol (≥9.0–15.0%) [3]. Furthermore, real-world studies involving patients with elevated HbA_1c_ levels show favourable impacts on metabolic control, with fewer hypoglycaemia events when using IDegLira [10, 11]. Also, the efficacy and safety of IDegLira are independent of gender. Therefore, no analysis was performed [3].

The efficacy of IDegLira was reflected in a higher TIR, reaching up to 80.6% vs 69.7% with MDI. This is consistent with findings in the literature, where these therapies achieve over 80% TIR [12, 13], along with a reduction in TAR. This finding is associated with the GLP-1 receptor agonist component (liraglutide), which enhances postprandial glucose management [14]. Another advantage of adding a GLP-1 receptor agonist to the treatment regimen is weight loss. In this study, patients treated with IDegLira lost approximately 2 kg, whereas the control group patients gained 1 kg. Similar results have been observed in other studies, which report stable weight or reductions in weight between 0.5 and 2 kg [11, 15–17]. This weight loss helps reduce insulin resistance and potentially lowers the risk of treatment failure, disease progression and associated complications [18].

In this study, IDegLira demonstrated superior outcomes compared with MDI in terms of combined clinical efficacy and safety outcomes, as assessed by CGM metrics. These findings are important and confirm the results of the TiREX study [13]. Subgroup analysis showed that the patients achieving substantial improvements in metabolic control, specifically a TIR >70%, were those with HbA_1c_ values >86 mmol/mol (>10%). Post hoc analysis of the DUAL VII study results suggests that patients with HbA_1c_ levels >58 mmol/mol (>7.5%) experience more pronounced improvements in metabolic control following intervention with IDegLira [9].

Adverse effects associated with IDegLira use were mainly gastrointestinal, with nausea and vomiting being more prevalent in this group. This is probably attributable to the liraglutide component, which is known for a higher incidence of gastrointestinal adverse symptoms compared with insulin [3, 4]. The literature describes an increase in gastrointestinal adverse symptoms, including nausea, vomiting and diarrhoea (OR 2.89), mainly during the medication titration phase [3]. However, none of the patients in this study discontinued therapy during follow-up.

One strength of this study is the use of CGM to assess safety and efficacy outcomes in relation to TIR, which correlates well with HbA_1c_ levels [8]. Moreover, this study expanded existing evidence on the benefits of use of a co-formulation treatment in patients with HbA_1c_ >86 mmol/mol (>10%). Also, the randomisation of patients ensures that other population characteristics such as age, sex, treatment prior to hospitalisation and cause of hospitalisation do not influence the results of the trial. However, the study has limitations, including a 5% loss of follow-up within the first 28 days. This reflects real-world patient behaviour, where some patients may not complete follow-up. Nevertheless, the study met the required sample size for analysis.

In conclusion, this study demonstrates that the IDegLira co-formulation offers superior safety and efficacy for treating type 2 diabetes patients with suboptimal metabolic control at hospital discharge. It provides a promising option for patients with high HbA_1c_ levels (>86 mmol/mol, >10%), who were traditionally considered candidates for MDI therapy.

## Supplementary Information

Below is the link to the electronic supplementary material.

ESM Tables (PDF 111 KB)

## Data Availability

The datasets generated or analysed for the current study are available upon reasonable request from the sponsor institution (Hospital Universitario San Ignacio).

## Abbreviations

CGM: Continuous glucose monitoring
GLP-1: Glucagon-like peptide-1
GMI: Glucose management indicator
IDegLira: Insulin degludec/liraglutide
MDI: Multiple daily insulin injections
TAR: Time above range
TBR: Time below range
TIR: Time in range
