# Supplementary material for: Insulin DEgludec/LIraglutide versus multiple daily insulin injections in the transition from hospital to outpatient management assessed by continuous glucose monitoring: the DELI transition trial
Source: Diabetologia. 2025 Aug 4;68(9):1899–907. doi: 10.1007/s00125-025-06446-y (PMC12361261; doi:10.1007/s00125-025-06446-y)
Supplement: Supplementary file 1 — ESM Tables (PDF 111 KB) [file 125_2025_6446_MOESM1_ESM.pdf]

ESM table 1. TITRATION OF INSULIN DOSES

MDI	IdegLira
• Insulin Glargine required in-hospital<br>• Insulin Aspart required in-hospital.<br>• Reduce 20% of the total daily dose administered the day before discharge.	• Insulin use prior to admission: start 16 units of IDegLira<br>• Without insulin use prior to admission: start 10 units of IDegLira

ESM Table 2. IDEGLIRA AND GLARGINE U100 TITRATION ALGORITHM (DUAL VII)

Average fasting blood glucose<br>mmol/L	Changing the dose<br>Insulin units (UI)
< 4	-2
4 – 5	0
>5	+2

ESM Table 3. PREPANDIAL ASPART TITLE ALGORITHM (DUAL VII)

Average fasting blood glucose (mmol/L)	Changing the dose<br><br>Insulin units (UI)	Rule for adjustment
< 4	-1	1 or more glucometry tests below the target
4 – 6	0	0-1 blood glucose above target,<br><br>no blood glucose below target
>6	+1	≥2 glucometry above target, no glucometry below target
